# Hybrid Adjuvant-Allergen H1sD2 Proteoforms Enhance Innate Immunity Activation via Distinct N-Glycosylation Profiles

**DOI:** 10.3390/cells14242008

**Published:** 2025-12-16

**Authors:** Zorana Lopandić, Maša Babović, Tina Ravnsborg, Marina Atanasković-Marković, Ole N. Jensen, Marija Gavrović-Jankulović

**Affiliations:** 1 Institute of Medical Chemistry, Faculty of Medicine, University of Belgrade, 11000 Belgrade, Serbia; 2 Department of Biochemistry, Faculty of Chemistry, University of Belgrade, 11158 Belgrade, Serbia; 3 Department of Biochemistry and Molecular Biology, University of Southern Denmark, 5230 Odense, Denmark; 4 Department of Allergology and Pulmonology, University Children’s Hospital, Faculty of Medicine, University of Belgrade, 11000 Belgrade, Serbia

**Keywords:** allergy, HDM, Der p 2, allergen-specific immunotherapy, adjuvants, N-glycans, hemagglutinin

## Abstract

**Highlights:**

**What are the main findings?**

- Five distinct N-glycosylated H1sD2 glycoforms were engineered and produced in *P. pastoris*
- H1sD2 glycoforms bind to DC-SIGN on M2 macrophages and modulate IL-10 and IFN-γ production in PBMCs from HDM-allergic donors

**What are the implications of the main findings?**

- N-glycan configuration influences innate immune activation and cytokine response
- Glycoengineering of allergens or therapeutic proteins may enhance immunomodulatory efficacy, with potential applications in allergy treatment and vaccine development

**Abstract:**

Novel adjuvants are key to making allergen-specific immunotherapy (AIT) safer and more effective. Their development is crucial for moving AIT into a new generation of precision medicine. N-glycosylation of protein antigens plays a pivotal role in modulating innate immune responses through enhanced recognition by pattern recognition receptors. New AIT vaccine strategies aim to exploit this by using innate-targeting adjuvants, modifying allergen structures, and routing early responses toward tolerance. Thus, we engineered five distinct N-glycosylated adjuvant configurations, composed of the receptor-binding domain of hemagglutinin (H1s) and Der p 2 (D2) allergen, to explore how glycan profile affects innate immune response for the application in therapeutic strategies for Type 1 hypersensitivity. Glycoengineered proteoforms produced in *Pichia pastoris* were structurally verified by mass spectrometry. Using M0 and M2 THP-1-derived macrophages, binding of all H1sD2 proteoforms to DC-SIGN was confirmed via confocal microscopy and flow cytometry. Stimulation of PBMCs with these proteoforms led to increased IL-10 and IFN-γ levels, indicating a shift toward regulatory immune responses. Notably, the M2 glycovariant elicited the strongest immunomodulatory signature, suggesting significant promise as a therapeutic candidate. These findings support the potential of glycoengineered allergen-adjuvant proteoforms to fine-tune innate immunity and improve the safety and efficacy of AIT.

## 1. Introduction

Approximately 30% of people worldwide suffer from allergies, which are chronic non-communicable illnesses caused by an inadequate immune response to harmless antigens [1,2]. House dust mites (HDMs) are a major source of respiratory symptom-causing inhalant allergens that pose a major global health concern [3,4,5]. One of the most clinically relevant allergens from HDMs is Der p 2, a potent inducer of allergic reactions [5,6,7].

Allergen-specific immunotherapy (AIT) is currently the only effective treatment for allergies, working by administering high doses of the causative allergen to induce long-lasting immune tolerance after therapy ends [8,9,10]. AIT modulates both innate and adaptive immune responses by gradually desensitizing mast cells and basophils to specific allergens, reducing hypersensitivity over time [2,11,12]. This process reduces allergen-specific T-helper 2 (Th2) cell activation, while promoting regulatory T- and B-cells and enhancing the production of specific blocking antibodies, such as IgG and IgA [13]. Although AIT offers long-term efficacy, its variable response across patients highlights the need for innovative approaches to enhance its effectiveness [14,15]. One promising strategy is the use of adjuvants, such as virus-like particles and glycans, which can boost Th1 cell activation and promote a more balanced immune response [5,16,17,18].

Glycans have been investigated as adjuvants to enhance immunogenicity and shift the immune response from Th2- to Th1-dominated pathways [19,20,21]. Changing the type, location, or number of N-linked glycans in an allergen′s glycosylation pattern can have a substantial impact on how the allergen interacts with immune cells and, consequently, the overall immune response [22,23,24,25,26].

C-type lectin receptors (CLRs) have a particularly important role in AIT due to their ability to recognize glycan structures on allergens and shape immune responses accordingly [27]. CLRs such as DC-SIGN and Dectin-1 can bind to specific carbohydrate moieties, making them key sensors for glycosylated allergens and engineered glycoproteins [28]. This interaction is crucial for directing antigen-presenting cells, especially dendritic cells and macrophages, toward a tolerogenic profile.

The biological activity of glycoproteins such as hemagglutinin (H), an important structural protein present on the surface of many viruses, including influenza, depends on its N-glycan composition [29,30,31,32]. Hemagglutinin plays a dual role in mediating viral entry and triggering immune recognition which promotes a Th1-type immune response [33]. This makes it a promising candidate for various immunological applications. Hemagglutinin, especially its receptor-binding domain (RBD), offers strong potential as a novel adjuvant for AIT. Its proven ability to induce robust immune responses, combined with widespread use in vaccine development, positions it as a promising scaffold for the design of glycoengineered hybrid adjuvant-allergen proteins [34,35,36].

In this study, we engineered and produced five distinct hybrid adjuvant-allergen proteoforms, H1sD2, by fusing the RBD of hemagglutinin with Der p 2. These proteoforms incorporate one conserved (N43) and three putative N-glycosylation sites (N81, N111, and N116) derived from the H1-RBD [36]. Using site-directed mutagenesis and *Pichia pastoris* expression system, we generated five specific H1sD2 glycoforms to explore how variations in glycan composition affect immune interactions. Our objective was to examine how these glycoengineered proteoforms engage with CLRs, particularly DC-SIGN expressed on macrophages, and to explore how these interactions affect the cytokine signature of peripheral blood mononuclear cells (PBMCs) from HDM-allergic individuals. This approach provides insight into how engineered glycosylation can modulate innate immune recognition and promote regulatory immune responses, offering a promising strategy for improving the efficacy of AIT.

## 2. Materials and Methods

### 2.1. Design of H1sD2 Hybrid Proteoforms

Five different recombinant H1sD2 hybrids were designed in silico using the sequence of receptor binding domain of hemagglutinin, H1_63–291_ (GenBank: ACQ99608) and Der p 2 allergen (GenBank: JN222809). The coding sequence was engineered to ensure that the conserved N-glycosylation at position N43 (N95 in H3 HA) was retained in each of the five glycoproteins. N-glycosylation was designed at N43 in WT glycoform, N43 and N111 (N158 in H3 HA) in M1 glycoform, N43 and Asp116 (N163 in H3 HA) in M2, N43 and Asp81 (N129 in H3 HA) in M3, and N43, N81, N111, and N116 in M4. The DNA sequences encoding the *h1sd2* constructs were synthesized by Synbio Technologies (Monmouth Junction, NJ, USA), and codon usage was optimized for the expression in *Pichia pastoris*. The synthesized *h1sd2* DNA fragments, as well as *d2* DNA fragment were cloned into the plasmid pPicZα-A vector (Invitrogen Thermo Fisher Scientific, Carlsbad, CA, USA) between the EcoRI and XbaI (Thermo Scientific, Waltham, MA, USA) sites for expression as a 6His-tagged protein on the C-terminus. *Escherichia coli* strain DH5α (Invitrogen Thermo Fisher Scientific, Carlsbad, CA, USA) was used as a host for the propagation of plasmid pPicZα-A (Invitrogen Thermo Fisher Scientific, Carlsbad, CA, USA) and *P. pastoris* Mut^S^ KM71H strain as host for expression of recombinant proteins.

### 2.2. Expression and Purification of the D2 and H1sD2

The plasmids were linearized with MssI (PmeI) (Thermo Scientific, Waltham, MA, USA) and then *P. pastoris* Mut^S^ strain KM71H was transformed by electroporation according to manufacturer’s protocols (EasySelect^TM^ *Pichia* Expression Kit, Invitrogen, Groningen, The Netherlands) and plated on YPDS (1% yeast extract, 2% peptone, 2% glucose, 18.2% sorbitol) agar plates containing 100 μg/mL Zeocin (Genaxxon Bioscience, Ulm, Germany). After transformation, *P. pastoris* clones were inoculated into 300 mL Buffered Glycerol-complex Medium, BMGY (1% yeast extract, 2% peptone, 1.34% yeast nitrogen base (YNB), 1% Glycerol, and 100 mM potassium phosphate; pH 6.0) at 28 °C until culture reaches an OD_600_ 2–6. Cells were harvested by centrifugation (5 min, 2000× *g*, Eppendorf centrifuge 5430R, Hamburg, Germany) at room temperature and further resuspended in Buffered Methanol-complex Medium, BMMY using 1/10 of the original culture volume. The expression was induced by 0.5% methanol in BMMY medium (1% yeast extract, 2% peptone, 1.34% YNB, 0.5% methanol, and 100 mM potassium phosphate; pH 6.0) every 24h for three days. After 72h, the cells were harvested by centrifugation (10 min, 3000× *g*, Eppendorf centrifuge 5430R, Hamburg, Germany). The supernatants (culture media), including proteins, were collected and dialyzed against 20 mM Tris-HCl (Merck, Darmstadt KGaA, Germany), 150 mM NaCl (Beta Hem, Belgrade, Serbia), pH 8.0 overnight. Thereafter, the samples were centrifuged (10 min, 12,100× *g*, Eppendorf 5453 MiniSpin Plus centrifuge, Hamburg, Germany) and loaded onto an Immobilized-Metal Affinity Chromatography (IMAC) column (HiTrap IMAC FF, 1mL with Co^2+^, GE Healthcare, Uppsala, Sweden), connected with an AKTA purifier (GE Healthcare, Uppsala, Sweden) with flow rate 1mL/min, respectively. The bound protein fraction was eluted with a 0–100% imidazole gradient with buffer A: 20 mM Tris-HCl, 150 mM NaCl, pH 8.0; buffer B: 20 mM Tris-HCl, 150 mM NaCl, pH 8.0, 0.3 M imidazole (Merck, Darmstadt KGaA, Germany). After IMAC, proteins were additionally purified by ion exchange chromatography on ANX Column (HiTrap ANX Fast Flow (High Sub), 1 mL, GE Healthcare, Uppsala, Sweden) on AKTA Purifier (GE Healthcare, Uppsala, Sweden). The bound protein fractions were eluted with a 0–100% NaCl gradient with buffer A: 20 mM Tris-HCl, pH 8.0; buffer B: 20 mM Tris-HCl, 500 mM NaCl, pH 8.0.

Even though the *P. pastoris* expression system typically produces recombinant proteins without endotoxins, all proteins were tested for endotoxin contamination using the Limulus amoebocyte lysate (LAL) assay (Thermo Fisher Scientific, Waltham, MA, USA) to ensure safety and quality, confirming that endotoxin levels were below 0.5 ng/mL (Supplementary Table S2).

### 2.3. Patients and Sera

The study was performed with the approval of the Ethics Committee of the University Children’s Hospital of Belgrade, Serbia (Approval No.: 017-16/23) under Serbian National guidelines (which follow the Declaration of Helsinki) for studies involving human subjects. Informed written consent was obtained before the study. Table S3 summarizes the clinical phenotype of the eight HDM-allergic patients who were included in the study.

### 2.4. Evaluation of IgE Reactivity in Immunoblot

The IgE reactivity of recombinant Der p 2 and H1sD2 glycoproteins was analyzed in immunoblot using serum from eight HDM-allergic persons. Purified Der p 2 and H1sD2 were resolved onto a 12% polyacrylamide gel under reducing conditions (in presence of 1% β-mercaptoethanol). For each analyzed protein, preparative SDS-PAGE was performed using a single wide well (~3 cm) loaded with the sample. After electrophoresis, the proteins were transferred onto a nitrocellulose membrane (Amersham™ Protran^®^, GE Healthcare, Chicago, IL, USA). This approach ensured that each membrane strip originated from the same gel lane and therefore contained an equal amount of the protein sample. Transfer of the proteins to the membrane was confirmed with Ponceau S staining and the membrane was cut into slices. The membranes were blocked with 2% bovine serum albumin (BSA) in 20 mM Tris-buffered saline with 0.2% Tween 20 (tTBS) pH 7.4 for 2 h and incubated with the patients’ sera in tTBS (dilution 1:10 = *v*:*v*) for 1 h. After washing, the membranes were incubated with monoclonal anti-human IgE-Alkaline Phosphatase (AP) antibody (Clone GE1, Sigma Aldrich, St. Louis, MO, USA) for 1 h. IgE reactivity of recombinant proteins was detected using nitro-blue tetrazolium chloride and 5-bromo-4-chloro-3′-indolyphosphate (BCIP/NBT, Serva, Heidelberg, Germany) in AP buffer (100 mM Tris-HCl pH 9.5, 100 mM NaCl, 5 mM MgCl_2_).

### 2.5. 3D Modeling

The sequence alignment of the five H1sD2 hybrid proteoforms was conducted by the online bioinformatics tool ClustalOmega (https://www.ebi.ac.uk/jdispatcher/msa/clustalo?stype=protein, accessed one 10 February 2025) [37]. The ColabFold (version 1.5.2) via AlphaFold 2 Server was used to create the H1sD2 protein models incorporating sequence information. Structural templates from the Protein Data Bank (PDB) were utilized to guide the modeling process [38].

### 2.6. Deglycosylation of Glycoproteins and SDS-PAGE Analysis

PNGase F (New England Biolabs, Ipswich, MA, USA) was used according to the non-denaturing protocol provided by the producer [39]. Before treatment with PNGase F, lyophilized samples D2, WT, M1, and M2 were reconstituted in Milli-Q water, while M3 and M4 were used directly in buffer without lyophilization. Briefly, 1–20 µg of protein was incubated with GlycoBuffer 2 (1×) and 2 µL of PNGase F at 37 °C overnight. After deglycosylation, the intact protein and PNGase-treated samples were analyzed on SDS-PAG Electrophoresis (NuPage™ Bis Tris Bolt™ 4–12%, Thermo Fisher Scientific, Waltham, MA, USA) under reducing condition with the addition of DTT (50 mM, Bolt^TM^ Sample Reducing Agent, Thermo Fisher Scientific, Waltham, MA, USA). Approximately 2 µg of each sample, either intact or deglycosylated, was loaded per lane, along with 4 µL of protein markers (10–140 kDa, Thermo Fisher Scientific, Waltham, MA, USA). Due to differences in the initial protein concentrations (0.5 mg/mL for D2, WT, M1, and M2; 0.1 mg/mL for M3; and 0.05 mg/mL for M4), larger sample volumes were required for the lower-concentration samples to ensure that 2 µg of protein was loaded onto the gel. Electrophoresis was performed at 160 V for 30 min, and bands were subsequently visualized using InstantBlue^®^ Coomassie Protein Stain (Abcam, Cambridge, UK).

### 2.7. Mass Spectrometry Analysis of H1sD2 Glycoproteins

#### 2.7.1. In-Solution Hybrid Proteoforms Digestion

Hybrid proteoforms were diluted to 1 μg/μL, reduced with 10mM DTT (Merck, Rahway, NJ, USA) for 30 min at 57 °C, alkylated with 20 mM iodoacetamide (Merck, Rahway, NJ, USA) for 30 min at room temperature in the dark, and the reaction was quenched by further addition of 10 mM DTT. Sequencing grade modified trypsin (Promega, Madison, WI, USA) and Endoproteinase Glu-C from *Staphylococcus aureus* V8 (Merck, Rahway, NJ, USA) were used for digestion. Each protein was digested by either trypsin alone (1:50 = *w*:*w*; overnight at 37 °C) or by a combination of Glu-C (1:20 = *w*:*w*; 4 h at 37 °C) and trypsin (1:50 = *w*:*w*; overnight at 37 °C). Digestion was stopped by the addition of formic acid, and peptides were desalted using a pipette tip packed with Poros R3 packing material (Thermo Fisher, Waltham, MA, USA) [40]. Glycopeptides were enriched using in-house packed tips with PolyHYDROXYETHYL A, 12 µm, 100 Å beads (PolyLC, Columbia, MD, USA) following the published protocol [41].

#### 2.7.2. In-Gel Hybrid Proteoforms Digestion

Both deglycosylated and glycosylated protein bands were excised from the PAA gel and digested following the published protocol [42]. Materials used were as described in the in-solution digestion section.

#### 2.7.3. Peptide and Enriched Glycopeptide LC-MS Data Acquisition

The analytical column (23 cm, 75 µm ID) used for LC-MS was packed with Reprosil-Pur 120 C18-AQ 3 µm packing material (Dr. Maisch GmbH, Ammerbuch, Germany) and connected to the Easy-nLC system coupled to Orbitrap Exploris (Thermo Scientific, USA). Solvent A (0.1% formic acid in MQ water) and B (95% Acetonitrile, 0.1% formic acid in MQ water) were used for elution. Enriched glycopeptides were separated using a 30 min gradient with 250 nL/min flow rate, and a shorter (23 min) gradient was used for in-gel digested samples at the same flow rate. Samples were analyzed in technical triplicates, and 2 pmol was injected in each replicate. The following method settings were used for enriched glycopeptides: spray voltage 2.1 kV, transfer tube temperature 275 °C, MS1 Orbitrap resolution 120,000. The top 10 precursors of charge 2–9 were selected for HCD (collision energy 35%) fragmentation in glycopeptide-enriched samples and recorded with MS2 Orbitrap resolution of 60,000. For in-gel digested samples, the highest intensity precursors of charges 2–6 were selected for unenriched samples for HCD fragmentation (collision energy 30%), and MS2 spectra were recorded with a nominal resolution of 45,000.

#### 2.7.4. Data Analysis

MS data was searched against a database containing *P. pastoris* (downloaded on 25 July 2022. from Uniprot.org) database to which the H1sD2 sequences were added along with a cRAP database (database of common contaminants compiled by the Global Proteome Machine Organization [43], using MS Amanda [44] and Sequest HT [45] nodes in Proteome Discoverer v 3.0 (Thermo Fisher Scientific, USA). MS1 tolerance was set to 10 ppm, and MS2 tolerance to 0.02 Da, and allowed FDR was 1%.

For enriched glycopeptide data processing, MSConvert (ProteoWizard 3.0) was used to convert the raw data files to .mzml data format [46]. GlycReSoft (version 0.4.25) was used for glycopeptide search [47]. Glycopeptide search tolerance was set to 5 ppm on MS and MS/MS levels. Glycopeptide spectra were manually inspected to confirm annotation.

### 2.8. Differentiation of THP-1 Cells into Macrophages

The THP-1 human monocyte cell line was purchased from the American Type Culture Collection (ATCC, Manassas, VA, USA). Cells were maintained in RPMI-1640 culture medium (Catalog number: A1049101, Thermo Fisher Scientific, Waltham, MA, USA) supplemented with 10% FBS (fetal bovine serum), 2 mM glutamine, 100 U/mL penicillin, 100 μg/mL streptomycin) at 37 °C in a humidified atmosphere of 5% CO_2_. The cells were differentiated into M0 macrophages by the addition of 100 ng/mL phorbol 12-myristate 13-acetate (PMA, Sigma-Aldrich, St. Louis, MO, USA) for 48 h. After differentiation, cells were washed twice with PBS and rested for 72 h in fresh media without PMA. To generate the M2 phenotype, after 48 h of PMA priming and 72 h rest, THP-1 differentiated macrophages were treated with 20 ng/mL of IL-4 for 48 h.

THP-1 cells were seeded in a 24-well plate (5 × 10^5^ cells/well), differentiated, and washed with PBS to characterize the macrophages. Subsequently, cells were incubated with accutase (PAN-Biotech GmbH, Aidenbach, Germany) for 20 min and collected into the FACS tubes (Falcon^®^ round bottom polystyrene tubes, Thermo Fisher Scientific, Hampton, NH, USA). Cell surface markers were stained following washing, per the manufacturer’s instructions. The following fluorescence-labeled antibodies were used: FITC-conjugated anti-CD53 (clone QA19A07, BioLegend, San Diego, CA, USA), FITC-conjugated anti-CD163 (clone GHI/61, BioLegend, San Diego, CA, USA), and PE-conjugated anti-CD206 (clone 19.2, Invitrogen Thermo Fisher Scientific, Waltham, MA, USA).

### 2.9. H1sD2 Hybrid Proteoforms Binding to THP-1 Differentiated Macrophages

The interactions between the H1sD2 hybrid proteoforms and the corresponding mannose-binding receptors on M0 and M2 THP-1-derived macrophages were examined using flow cytometry. The H1sD2 glycoproteins and Der p 2 were previously labeled using Fluorescein Isothiocyanate (FITC; Sigma Aldrich, St. Louis, MO, USA) following the manufacturer’s instructions. In brief, protein samples were prepared in fresh 0.1 M sodium carbonate buffer, pH 9. Very slowly, 1 mg/mL of FITC solution (DMSO: buffer, *v*:*v* = 1:20) was added in 5 μL aliquots while stirring gently. Following an 8-h dark incubation period at 4 °C, 50 mM of NH_4_Cl was added, and the mixture was then incubated for an additional 2 h at 4 °C. Labeled proteins were separated from unbound FITC by gel filtration on Bio-Gel P-30 matrix (Bio-Rad Laboratories, Hercules, CA, USA).

THP-1 cells were seeded in a 24-well plate (5 × 10^5^ cells/well) and differentiated into M0 and M2 phenotypes. FITC-labeled Der p 2 and H1sD2 hybrid proteoforms (500 nM each) were incubated with the cells for two hours at 37 °C and 5% CO_2_. Following three rounds of washing, the cells were detached using Accutase (PAN-Biotech GmbH, Aidenbach, Germany), washed once again, and then resuspended in 500 μL of PBS containing 0.2% BSA/NaN_3_ for flow cytometry analysis. A total of 10,000 cells were analyzed using BD FACSCalibur^TM^ (BD Biosciences, San Jose, CA, USA). BD CellQuest^TM^ Pro Software version 5.1 (BD Biosciences, San Jose, CA, USA) was used to analyze the raw data, and the percentage of positive cells was reported.

### 2.10. Confocal Microscopy

M2 differentiated macrophages were stained with FITC-labeled H1sD2 glycoproteins and PerCP-Cyanine 5.5 anti-human CD209 (DC-SIGN) monoclonal antibody (clone 9E9A8, BioLegend, San Diego, CA, USA) to test H1sD2 glycoproteins binding to DC-SIGN receptor specific for high mannose structures on viral hemagglutinins. THP-1 cells were seeded in complete RPMI-1650 medium on glass slides in a 24-well plate (1.75 × 10^5^ cells/well) and differentiated into M2 macrophages as previously described. Following a PBS wash, cells were incubated for two hours (37 °C, 5% CO_2_) with FITC-labeled Der p 2 or H1sD2 glycoproteins, respectively. Afterward, cells were washed three times, and nonspecific interactions were blocked with 1% human serum albumin (HSA, Serva GmbH, Heidelberg, Germany) in PBS (10 min, room temperature). Then, the cells were washed, incubated for 20 min at 4 °C with anti-human DC-SIGN-Per CP-Cy 5.5 antibody, washed again and fixed for 15 min at room temperature to glass slides with 4% paraformaldehyde in 1% PBS.

After washing with PBS, cells were analyzed by a confocal microscope (Leica TCS SP5 II, Leica Microsystems, Wetzlar, Germany). Images were acquired with 64× magnification of the objective.

### 2.11. Sandwich ELISA-Based Detection of Proteoform-Induced Cytokine Signatures in PBMCs

PBMCs were isolated from five HDM allergic donors. Whole blood (EDTA anticoagulant) from the donors was provided by the University Children’s Hospital (Table S3, patients 1, 2, 4, 5 and 8). Blood was transferred from the collecting tube to a vial and an equal volume of 1 × PBS was added (*v*:*v* = 1:1). Samples were gently mixed and added over the Histopaque^®^-1077 (Sigma Aldrich, Poznań, Poland) by pipetting slowly, and centrifuged without brake (450× *g*, at 20 °C for 35 min). The PBMC layers were carefully removed from the tube and transferred to a new conical tube. The PBMCs were washed twice with 1× PBS. After centrifugation (300× *g*, at 4 °C for 15 min), the cells were resuspended in 1mL of RPMI 1640 medium supplemented with 1% Penicillin/Streptomycin and 1% L-Glutamine (Sigma-Aldrich, St. Louis, MO, USA), counted and diluted to 1 × 10^6^ cells per mL, and 250 μL per well was seeded into the 96-well plate for treatment. Der p 2 and H1sD2 glycoproteins were added to the cells at equimolar concentrations (500 nM in RPMI 1640 without FBS, 37 °C, 5% CO_2_) for 48 h. IL-4, IL-10, and IFN-γ were measured in the culture supernatant with the ELISA kits (BioLegend, San Diego, CA, USA) whose sensitivities were 3.9 pg/mL, 3.9 pg/mL, and 7.8 pg/mL, respectively.

### 2.12. Statistical Analysis

Data was expressed as mean values ± SD. Statistical analyses were conducted using Prism 9 (GraphPad, La Jolla, CA, USA). The statistical significance was evaluated by one-way or two-way analysis of variance (ANOVA) with a Bonferroni test. The results were considered statistically significant if *p* < 0.05. All experiments were independently performed at least 3 times in duplicate/triplicate.

## 3. Results

### 3.1. Design, Expression and Purification of H1sD2 Hybrid Proteoforms

**Figure 1:**
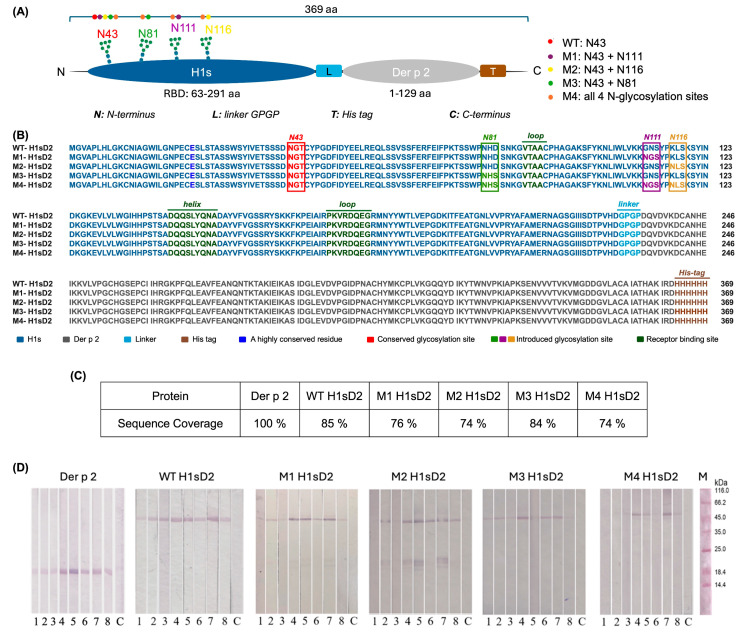
Schematic representation, sequence alignment, mass spectrometry amino acid sequence coverage and IgE reactivity of Der p 2 and five hybrid adjuvants-allergen H1sD2 proteoforms. (**A**) Schematic representation of the hybrid adjuvant–allergen H1sD2 proteoforms. Each H1sD2 proteoform consists of the receptor-binding domain of influenza hemagglutinin (H1s) fused to the major house dust mite allergen Der p 2. The H1s domain was engineered to contain different numbers and positions of N-glycosylation sites. The M4 variant carries all four introduced N-glycosylation sites, while other proteoforms contain one (WT) or two glycosylation sites (M1, M2, M3). (**B**) Multiple Sequence Alignment of five H1sD2 glycoprotein sequences using Clustal Omega (https://www.ebi.ac.uk/jdispatcher/msa/clustalo, accessed on 10 February 2025). Sequence variations are highlighted in different colors, with a legend included in the figure. (**C**) Percent of amino acid sequence coverage of in-gel digested samples (Proteome Discoverer 3.0). (**D**) Der p 2 and H1sD2 hybrids bind IgE from sera of HDM-allergic individuals. 1–8: patient samples; C—control for secondary antibodies (monoclonal anti-human IgE-AP); M—molecular weight markers. The levels of HDM-specific IgE in individual sera were measured using ImmunoCAP and are presented in Table S3.

Five hybrids comprising the HDM allergen Der p 2 and the RBD of the influenza hemagglutinin with different N-glycan sites were generated (H1sD2 proteoforms) in silico and produced in the *P. pastoris.* To facilitate visualization of the designed constructs, a schematic representation of the hybrid adjuvant-allergen H1sD2 proteoforms was prepared, illustrating the position and number of N-glycosylation sites in each variant (Figure 1A). The multiple sequence alignment of the generated H1sD2 glycoproteins is presented in Figure 1B, providing a detailed comparison of their amino acid sequences.

Der p 2 and five H1sD2 hybrid glycoproteins were produced as C-terminal 6-His-tagged molecules in the *P. pastoris* expression system. They were then isolated by immobilized metal affinity chromatography and ion-exchange chromatography. In SDS-PAGE analysis under reducing conditions, the purified H1sD2 proteins showed bands with an approximate molecular weight of 48 kDa, (Figure S1, Line 1). Among the H1sD2 glycoforms, M4 was produced at lower yields, as reflected by the weaker band intensities on the gel (Figure S1). This can be attributed to its extensive N-glycosylation (four N-glycosylation sites). Despite several optimization attempts, the expression and recovery of M4 remained low, resulting in a reduced protein concentration in this sample. Nevertheless, the protein band is clearly identifiable even though diffused, which correlates with literature data that extensive glycosylation also likely contributed to the diffuse appearance of the bands [48]. After treatment with PNGase F, which cleaves N-glycans from the glycoprotein surface [49,50], the H1sD2 proteins displayed a noticeable reduction in apparent molecular weight on the gel, illustrating the contribution of N-glycosylation to their overall molecular mass (Figure S1, Line 2). Due to differences in sample preparation and protein concentrations, larger volumes of the lower-concentration samples (M3 and M4) were used, resulting in a greater total amount of PNGase F and, consequently, a more intense PNGase F band on the SDS-PAGE. Furthermore, variations in the ionic strength of the samples are known to affect band sharpness [51], which likely explains the more diffuse bands observed for M3 and M4. Densitometric analysis using protein markers allowed quantification of the approximately 3–4 kDa shifts observed after endoglycosidase treatment, confirming the removal of N-glycans (Table S1). Additionally, it is well known phenomenon that glycoproteins show reduced electrophoretic migration in SDS-PAGE due to inefficient detergent binding to glycans, resulting in higher molecular mass in gel then predicted [52,53]. For Der p 2, which showed an apparent molecular weight of approximately 17 kDa (Figure S1), the presence of disulfide bridges hampers its migration under both non-reducing and reducing conditions. The protein exhibits an abnormal electrophoretic mobility, likely reflecting its unique amino acid composition and compact tertiary structure [53].

### 3.2. IgE Reactivity Was Preserved in All Five H1sD2 Hybrid Proteoforms

An immunoassay was performed using sera from HDM-allergic patients to check the IgE immunoreactivity of H1sD2 glycoproteins and Der p 2 (Figure 1D). Importantly, in accordance with the sIgE values determined in ImmunoCAP, patient sera No. 4, 5, and 7 exhibited strong IgE reactivity with Der p 2 and all H1sD2 proteoforms, whereas the remaining sera displayed weaker IgE reactivity due to their low sIgE levels (Table S3). The immunoreactivity is visible even with M4 proteoform that was expressed with lower yield. These results suggest that the pattern of IgE reactivity found for Der p 2 was preserved in all five H1sD2 hybrids.

### 3.3. Three-Dimensional Models of H1sD2 Hybrid Proteoforms

**Figure 2:**
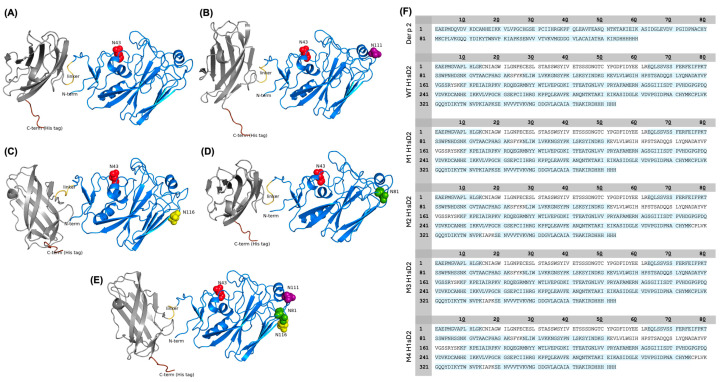
In silico structural prediction and sequence coverage of five H1sD2 hybrid proteoforms. (**A**–**E**) AlphaFold predicted 3D models of five H1sD2 hybrid proteoforms, generated via the AlphaFold Server. Models include: (**A**) WT H1sD2; (**B**) M1 H1sD2; (**C**) M2 H1sD2; (**D**) M3 H1sD2; (**E**) M4 H1sD2. The Der p 2 domain is shown in gray, while the H1-RBD is depicted in blue. The GPGP linker and 6His-tag are represented in yellow and brown, respectively. Engineered N-glycosylation sites are highlighted in red (N43), green (N81), pink (N111), and yellow (N116). (**F**) Sequence coverage maps of Der p 2 and H1sD2 proteins. Amino acid residues shown in blue were covered by a high-confidence peptide identification.

Amino acid sequences of Der p 2 and receptor binding domain of H1 proteins were extracted from the NCBI database (https://www.ncbi.nlm.nih.gov/). Using the Protein Data Bank (PDB) as a structural template source, in silico 3D models of H1sD2 hybrid proteoforms were generated using the AlphaFold Server (Figure 2A–E).

### 3.4. Mass Spectrometry Analysis of H1sD2 Hybrid Proteoforms

Peptide mapping and sequencing by mass spectrometry (LC-MS/MS) provided high amino acid sequence coverage (Figure 1C and Figure 2F). Analysis of enriched glycopeptides showed that the endogenous N-glycosylation site at N43 in WT H1sD2 was occupied in all H1sD2 glycoproteins, and novel engineered glycosylation sites in M1, M2, and M3 were also confirmed. In the M4 variant, both N43 and N81 were glycosylated while N111 and N116 were to be singularly glycosylated. A summary of detected glycopeptides is shown in Table 1, while the complete list of identified peptides is provided in Table S4, and examples of MS/MS spectra for each identified glycopeptide are shown in Supplementary Materials (Figures S2–S15).

### 3.5. All Five FITC-Labeled H1sD2 Hybrid Proteoforms Bind to M0 and M2 THP-1-Derived Macrophages

**Figure 3:**
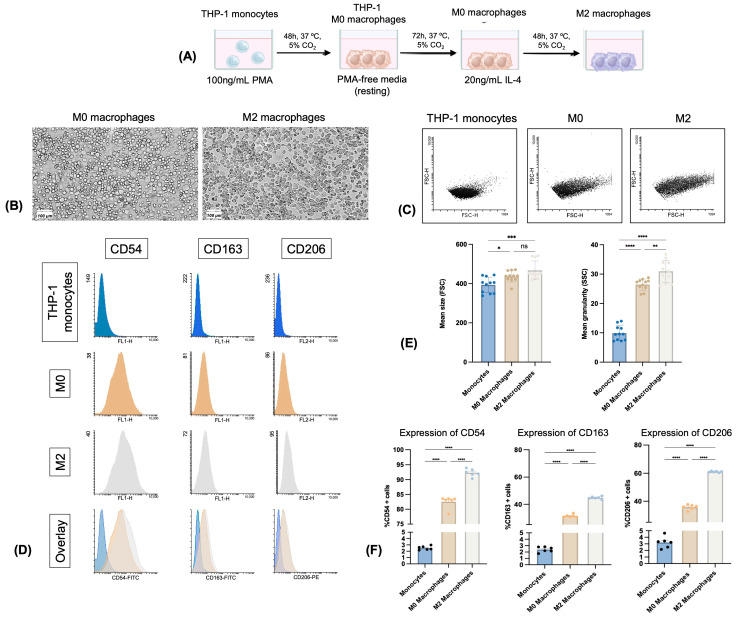
Flow cytometry analysis of THP-1 monocytes and differentiated M0 and M2 macrophages. (**A**) Schematic representation of the differentiation process of THP-1 monocytes into M0 and M2 macrophages; (**B**) Typical morphology of M0 and M2 macrophages observed by MilliCell DCI imaging system (scale: 100 μm); (**C**) dot plots of monocytes, M0 and M2 macrophages; (**D**) flow cytometry histograms of surface marker expression: CD54, CD163, and CD206 in THP-1 monocytes, M0 and M2 macrophages; (**E**) differences in cell size and granularity based on forward and side scatter parameters; (**F**) quantitative analysis of CD54, CD163, and CD206 surface markers expression. Data are presented as mean ± standard deviation (SD) of three independent experiments, each performed in duplicate. Statistical analysis was performed by Bonferroni’s test. Significant differences between groups are marked with: * *p* < 0.05, ** *p* < 0.01, *** *p* < 0.001, **** *p* < 0.0001, ns—not significant.

The morphological differences between THP-1-derived monocytes and macrophages were verified by FACS, comparing their size and granularity (Figure 3). Additionally, the differentiation of THP-1 monocytes into M0 and M2 macrophage phenotypes was confirmed by the expression of the surface markers CD206 (MR, mannose receptor), CD163 (high-affinity scavenger receptor), and CD54 (ICAM-1, intercellular adhesion molecule 1) (Figure 3).

**Figure 4:**
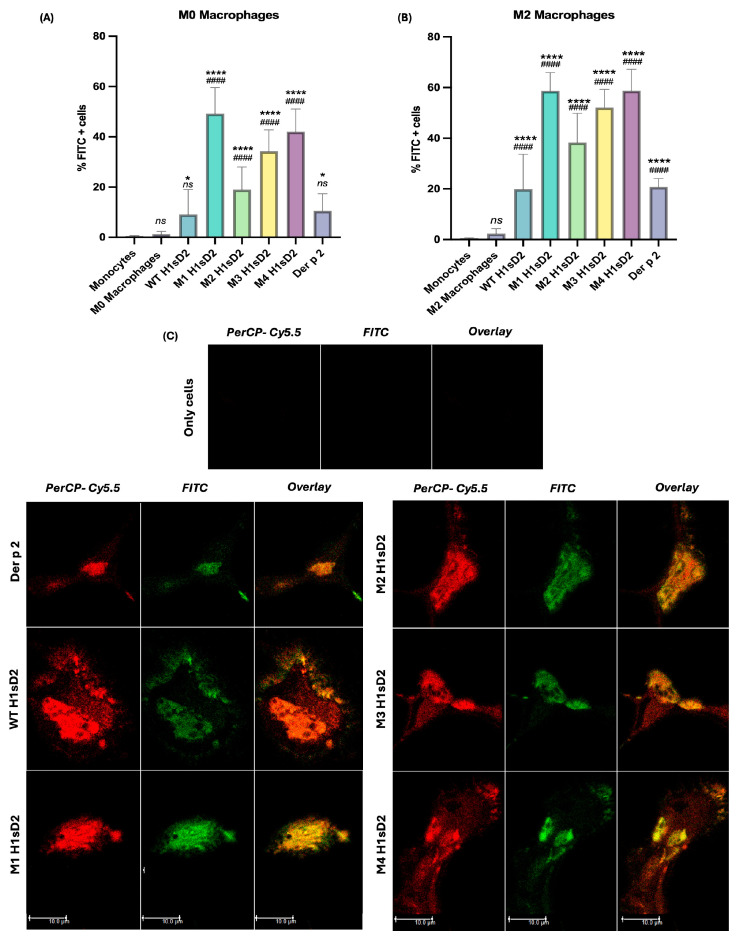
All five FITC-labeled H1sD2 hybrid proteoforms bind to M0 and M2 THP-1 derived macrophages. (**A**,**B**) Flow cytometry analysis confirms specific binging of FITC-labeled H1sD2 proteoforms to (**A**) M0 and (**B**) M2 polarized THP-1-derived macrophages. Data are presented as mean ± SD from three independent experiments. Statistical analysis was performed by Bonferroni’s test. Significant differences between groups are marked with: * *p* < 0.05, **** *p* < 0.0001 compared to monocytes and #### *p* < 0.0001 compared to M0/M2 macrophages, ns—not significant. (**C**) Confocal microscopy further validates the interaction between H1sD2 proteoforms and DC-SIGN receptor on M2 macrophages. DC-SIGN was visualized in red using PerCP-Cy5.5-conjugated anti-DC-SIGN antibodies, while FITC-labeled H1sD2 proteoforms are shown in green. The overlaid figures are also presented in the third column. Images were acquired using 64× objective magnification; scale bar = 10 μm. All experimental details can be found in Section 2.

Subtle differences in H1sD2 glycoproteins binding to the surface of M0 and M2 differentiated macrophages was detected by flow cytometry (Figure 4A,B). The trend line of glycoproteins binding was similar for both cell phenotypes. The order in which the proportion of FITC-positive cells increased was M2 H1sD2, M3 H1sD2, M4 H1sD2, and M1 H1sD2 glycan configuration, respectively (Figure 4). WT glycoprotein binding was similar to that of the Der p 2 allergen. The greater proportions of FITC-positive cells in the M2 phenotype as opposed to M0 can be explained by the increased surface expression of various mannose-binding receptors on M2 cells [54,55,56].

### 3.6. Confocal Microscopy Confirms H1sD2 Binding to DC-SIGN Receptor on M2 Macrophages

To confirm the binding of H1sD2 glycoproteins to mannose-specific pattern recognition receptors (PRRs), M2 macrophages were incubated with H1sD2-FITC glycoproteins along with the use of anti-DC-SIGN-PerCP-Cy5.5. The stained cells were analyzed by confocal microscopy. The results suggest that the H1sD2 hybrids decorated with mannose residues bind to M2 macrophages through interaction with the mannose-specific DC-SIGN receptor (Figure 4C, Figure S16).

### 3.7. H1sD2 Hybrid Proteoforms Induce Distinct PBMCs Cytokine Signatures

**Figure 5:**
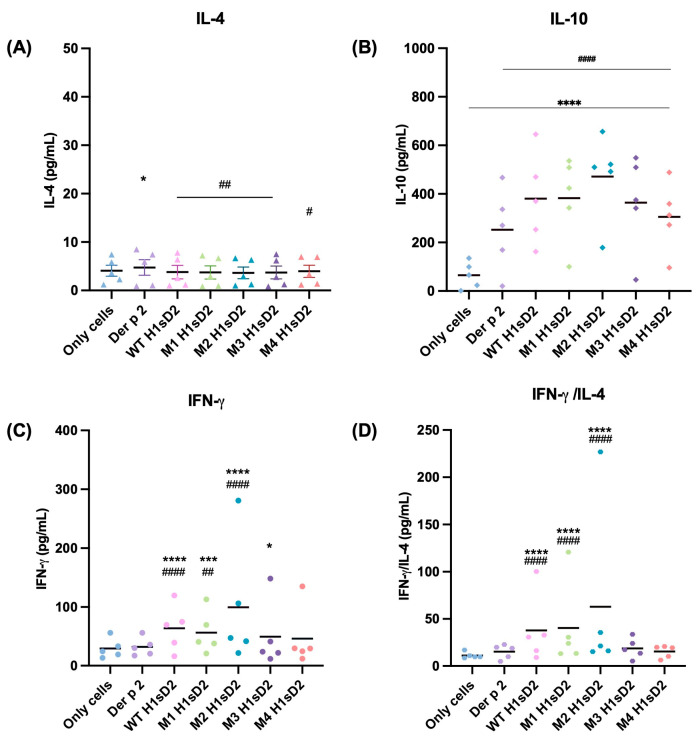
H1sD2 hybrid proteoforms induce distinct cytokine signatures in PBMCs from allergic individuals. PBMCs were isolated from the blood of HDM allergic donors and stimulated for 48 h with Der p 2 or individual H1sD2 glycoproteins. Two biological replicates of each sample were analyzed in duplicate. Cytokine secretion was quantified by sandwich ELISA for (**A**) IL-4, (**B**) IL-10, (**C**) IFN-γ, and (**D**) IFN-γ/IL-4 ratio as a Th1/Th2 balance indicator. Data are presented as mean values. Statistical significance was assessed by two-way ANOVA followed by Bonferroni’s test. Significant differences between groups are marked with: * *p* < 0.05, *** *p* < 0.001, **** *p* < 0.0001 compared to untreated control cells and # *p* < 0.05, ## *p* < 0.01 #### *p* < 0.0001 compared to Der p 2.

The immunomodulatory capacity of H1sD2 glycoproteins were tested in PBMCs from five allergic patients to HDM (Table S3, samples 1, 3, 4, 5 and 8). Due to factors such as donor availability, health status, logistical limitations, and variability among patients, not all individuals allergic to HDM could participate in this study. PBMCs were isolated from available plasma donors and IL-4, IL-10 and IFN-γ levels were measured by sandwich ELISA after 48 h of stimulation. The level of IL-4 secreted by PBMCs after treatment was relatively low. In contrast to only cells, Der p 2 significantly increased IL-4 production (Figure 5A). The secretion of IL-10 was statistically significant for all of the H1sD2 glycoproteins compared to only cells and Der p 2 (*p* < 0.0001, Figure 5B). The secretion of IFN-γ and IFN-γ/IL-4 ratio was statistically significant compared to only cells and Der p 2 for WT, M1, and M2 H1sD2 glycoproteins (Figure 5C,D).

## 4. Discussion

Allergies are a major global health concern that impacts the quality of life for hundreds of millions of people worldwide [1,2]. Although there are a variety of challenges with safety, effectiveness, and the length of therapy, AIT is a promising strategy that seeks to alter the underlying Th2 immune response to allergens and offer long-term relief. Adjuvants play a crucial role in enhancing adaptive immune responses by targeting innate immune cells and PRRs [21,57]. Additionally, adjuvants promote the expression of antigen-presenting molecules and costimulatory signals (e.g., CD40, CD80/CD86) on APCs, which is essential for T cell activation and immune memory formation [21,58].

Among emerging adjuvants, glycans are gaining attention for their potential to boost immune responses [5,16,17,18,19,59]. Glycans, which are critical for viral stability, pathogenicity, immunogenicity, and immune evasion, are present on viral envelope proteins. These glycans undergo changes in response to immune pressure, providing insights into their strategic inclusion in the design of immunogens [60,61,62].

In our study, five distinct H1sD2 hybrid proteoforms configurations were successfully produced in the *P. pastoris* expression system, a methylotrophic yeast platform known for its high cell density growth and efficient genetic manipulation [63]. This system is particularly advantageous for producing eukaryotic proteins with proper post-translational modifications, including N-linked glycosylation. *P. pastoris* typically attaches high-mannose type N-glycans, predominantly ranging from Man_8_GlcNAc_2_ to Man_14_GlcNAc_2_, although minor extensions with additional mannose residues or terminal α-1,2–linked mannoses can also occur [64,65]. The *P. pastoris* strain KM71H (aox1: ARG4, arg4) was employed for production due to its ability to express glycosylated, biologically active recombinant proteins [66,67]. The system’s success in producing glycoproteins is exemplified by its use in large-scale production of recombinant vaccines such as hepatitis B surface antigen (rHBsAg) [68] and the HPV vaccine [69].

Mass spectrometry analysis confirmed the amino acid sequences of the H1sD2 glycoproteins, with the glycosylation sites (N43, N81, N111, and N116) occupied by predominantly Man9—Man11 glycans, which is in line with previous reports [70]. Consistently, PNGase F treatment followed by SDS-PAGE revealed a clear reduction in apparent molecular mass, confirming that the observed mass heterogeneity among the H1sD2 variants resulted from N-linked glycosylation. Importantly, deglycosylation produced the expected shifts in protein size and electrophoretic mobility. Taken together, these observations justified prioritizing mass spectrometry for a more comprehensive and quantitative analysis of glycosylation. The IgE reactivity of Der p 2 and the five H1sD2 hybrids were confirm with sera from patients with HDM allergy, suggesting that the B cell epitopes on Der p 2 were preserved.

We also assessed the immunological effects of five H1sD2 hybrid proteoforms configurations by treating PBMCs isolated from HDM-allergic patients. A significant increase in IL-10 and IFN-γ levels was observed, signaling a shift toward a Th1 immune response. Both IL-10 and IFN-γ play complementary roles in allergen-specific immunotherapy by promoting immune tolerance. IL-10 facilitates the transition from a Th2 to Th1 response [71,72], encourages the production of allergen-specific IgG [2,73,74], and modulates pro-inflammatory cytokines [73,75]. IFN-γ further supports this shift by inhibiting Th2 cytokines, thereby fostering a more balanced immune response [76]. This cytokine modulation is crucial for reprogramming the immune system to a state of tolerance, which is the ultimate goal of AIT.

Furthermore, using the DC-SIGN as a model of the mannose-specific pattern recognition receptor on M2 macrophages, we examined the impact of N-linked glycan configurations on H1sD2 hybrids, particularly in relation to their interaction with the receptor. The THP-1 cell line is a suitable and reliable in vitro model for studying monocyte and macrophage responses, due in large part to its similarity to human peripheral blood mononuclear cell (PBMCs) derived monocytes and macrophages [77]. DC-SIGN, which is expressed on myeloid dendritic cells and macrophages [72,78], has been shown to interact with specific carbohydrate structures [79,80]. We confirmed that all five H1sD2 glycoproteins bind to DC-SIGN receptor on THP-1 macrophages, with the M2 macrophage phenotype showing higher binding due to increased expression of mannose-specific receptors, as demonstrated by flow cytometry analysis. Binding of H1sD2 glycoprotein configurations for M2 macrophages is visualized by confocal microscopy. Among the designed H1sD2 glycoproteins, M2 glycoform showed the best performance in terms of induced level of secreted IFN-γ/IL-4, and IL-10.

Despite the promising immunomodulatory effects observed, a limitation of the current design is the IgE-binding capacity of allergen-derived H1sD2 glycoproteins, which may restrict their translational potential. Additionally, the relatively low yield of recombinant proteins (around 20–50 mg/L) represented additional challenge to be solved, particularly for M4 glycoform, likely due to the presence of four introduced N-glycosylation sites and the high-mannose glycans profile produced in *P. pastoris*. Future work will focus on employing hypoallergenic Der p 2 isoforms with preserved T cell epitopes to preserve the immunomodulatory effects while minimizing IgE reactivity, testing the glycoforms in animal models, and performing detailed structural analyses. Additional experiments will also evaluate macrophage activation profiles, including cytokine secretion and activation marker expression, to further elucidate the mechanisms of glycan-mediated immunomodulation.

## 5. Conclusions

In conclusion, Der p 2 and five H1sD2 hybrid proteoforms were successfully produced in the *P. pastoris* expression system. All proteoforms retained structural integrity and distinct glycosylation profiles, with full N-glycan site occupancy confirmed by mass spectrometry. The binding of all proteoforms to receptors on M0 and M2 THP-1-derived macrophages, particularly to DC-SIGN on M2 phenotype, was confirmed using confocal microscopy and flow cytometry. Additionally, immunoblotting with sera from allergic patients demonstrated that IgE-binding epitopes were preserved across all glycoproteins, which was important for evaluation of the proof-of concept. PBMC stimulation from HDM-allergic donors revealed increased levels of IL-10 and IFN-γ, especially with the M2 H1sD2 variant. Despite the limited sample size, these results highlight M2 H1sD2 as a promising AIT candidate and support the broader potential of glycoengineered allergen-adjuvant proteoforms as novel adjuvants for safer and more effective immunotherapy.

## Figures and Tables

**Table 1:** Glycopeptide analysis of H1sD2 glycoproteins. Cysteine residues were alkylated and are shown in orange, and theoretical N-glycosylation sites are shown in blue.

Protein	AsnX	Peptide Sequence
WT H1sD2	Asn43	TSSSDNGTCYPGDFIDYEE
M1 H1sD2	Asn43	TSSSDNGTCYPGDFIDYEE
	Asn111	NGSYPKLSK
M2 H1sD2	Asn43	TSSSDNGTCYPGDFIDYEE
	Asn116	KGNSYPNLSK
M3 H1sD2	Asn43	TSSSDNGTCYPGDFIDYEE
	Asn81	TSSWPNHSSNK
M4 H1sD2	Asn43	TSSSDNGTCYPGDFIDYEE
	Asn111 or Asn116	NGSYPNLSK
		KNGSYPNLSK
	Asn81	TSSWPNHSSNK

## Data Availability

The data supporting the findings of this study are available from the corresponding authors upon reasonable request.

## Supplementary Materials

The following supporting information can be downloaded at: https://www.mdpi.com/article/10.3390/cells14242008/s1, Figure S1: SDS-PAGE of Der p 2 and H1sD2 proteins; Figures S2–S15 show examples of annotated glycopeptide spectra detected in H1sD2 proteins. Figure S16: Confocal microscopy analysis of double-stained M2 macrophages with FITC-labeled H1sD2 and anti-DC-SIGN antibody; Table S1: Theoretical and experimental determination of protein molecular masses, calculated from amino acid sequences (ProtParam) and estimated from SDS-PAGE band positions by densitometric analysis (ImageJ); Table S2: Endotoxin determination in protein samples using LAL assay; Table S3: Clinical phenotypes of study participants; Table S4: List of identified peptides.
