# Supplementary material for: Hybrid Adjuvant-Allergen H1sD2 Proteoforms Enhance Innate Immunity Activation via Distinct N-Glycosylation Profiles
Source: Cells. 2025 Dec 16;14(24):2008. doi: 10.3390/cells14242008 (PMC12731569; doi:10.3390/cells14242008)
Supplement: Supplementary file 1 [file cells-14-02008-s001.zip › Supplementary Materials_Cells.pdf]

SDS-PAG Electrophoresis profile of H1sD2

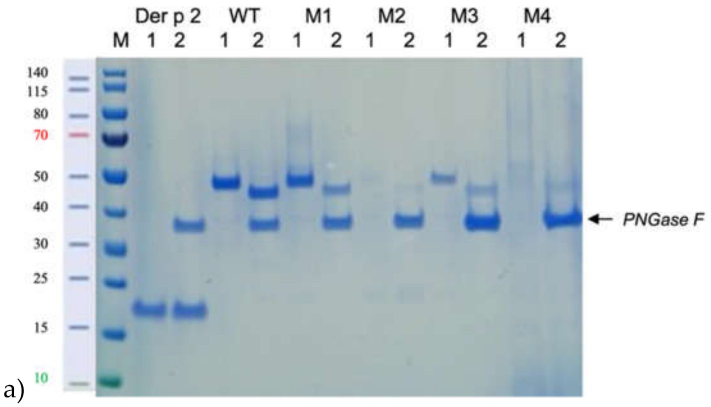

a)

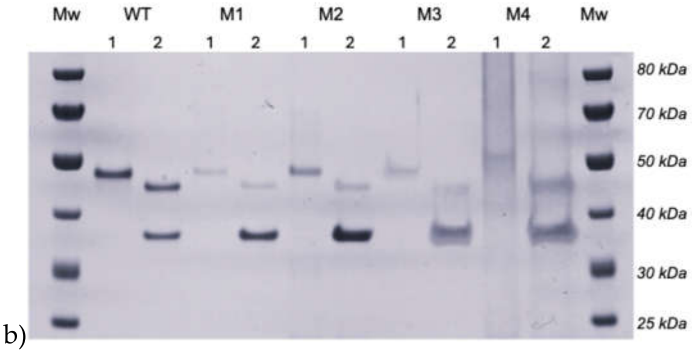

b)

Figure S1: SDS-PAGE of Der p 2 and H1sD2 proteins. (a) Analysis of Der p 2 and five H1sD2 variants. (b) Analysis of all H1sD2 variants. M – protein markers (10–140 kDa); 1 – intact protein; 2 – deglycosylated protein. The band at ~36 kDa corresponds to PNGase F.

Figures below (S2–S15) show examples of annotated glycopeptide spectra detected in H1sD2 proteins.

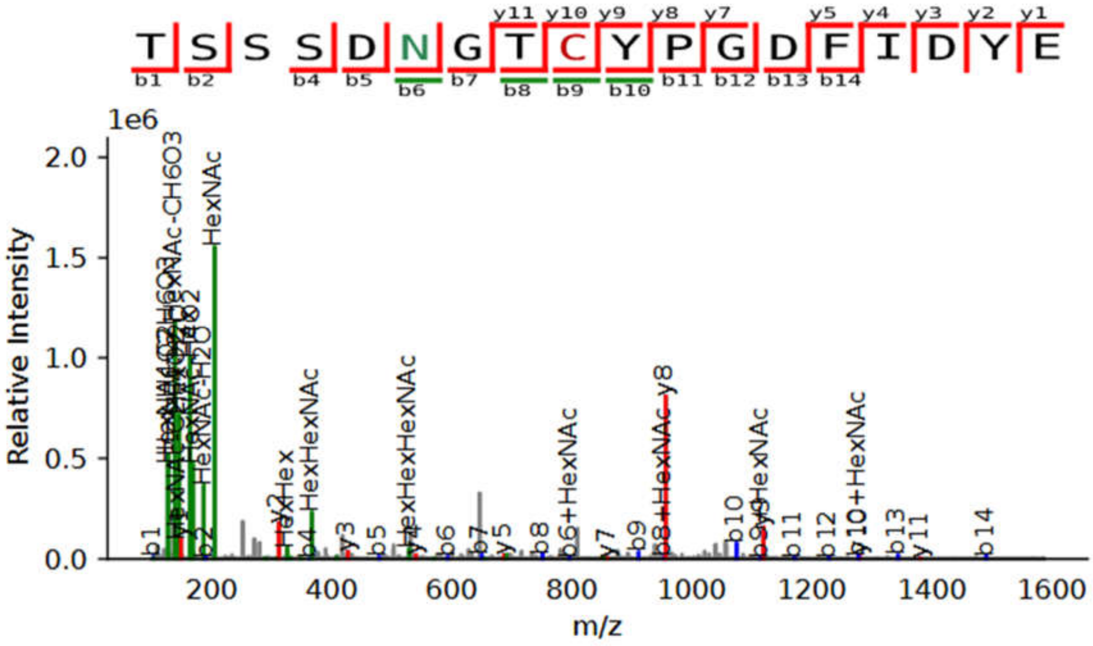

Figure S2. Annotated MSMS spectrum of WT H1sD2 peptide TSSSD**N**GTCYPGDFIDYE with Man10 glycan

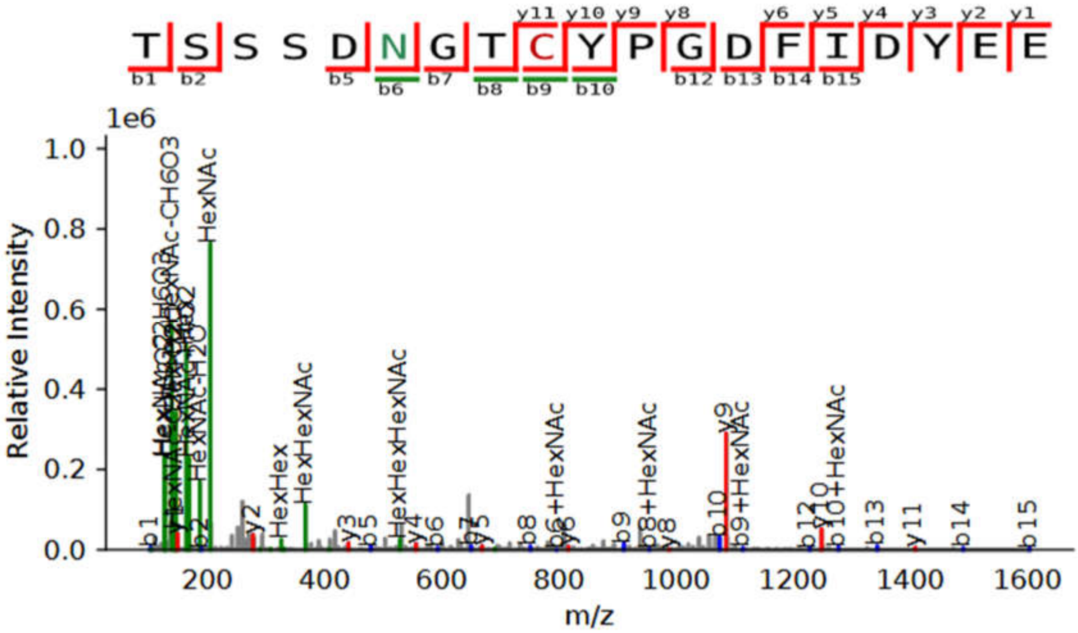

Figure S3. Annotated MSMS spectrum of WT H1sD2 peptide TSSSD**N**GTCYPGDFIDYEE with Man10 glycan

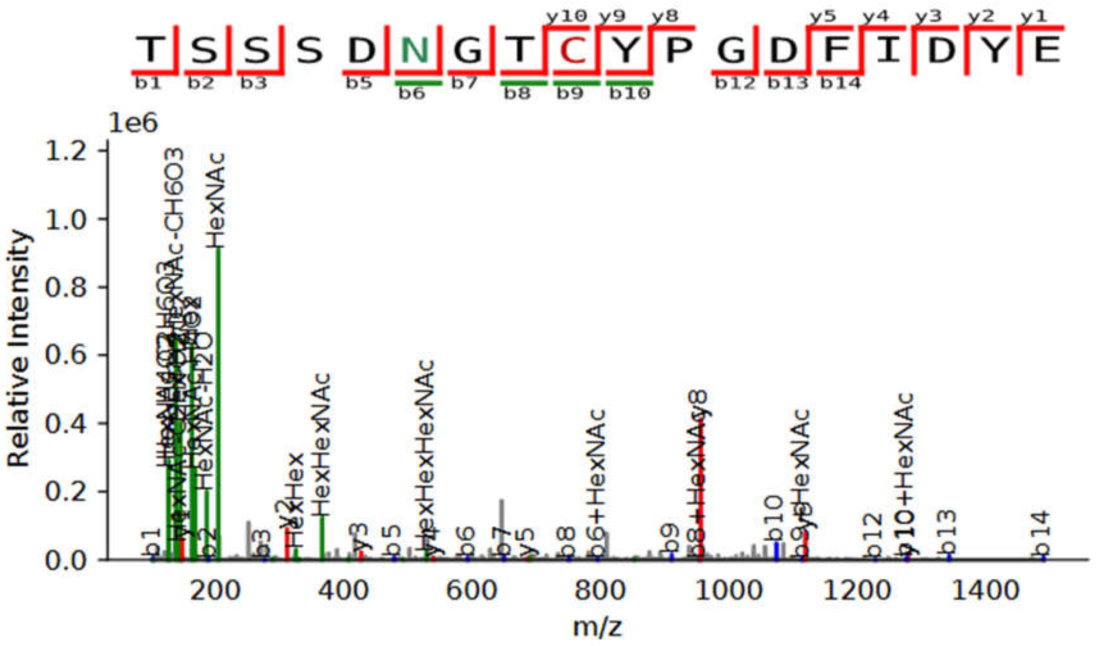

Figure S4. Annotated MSMS spectrum of M1 H1sD2 peptide TSSSD**N**GTCYPGDFIDYE with Man10 glycan

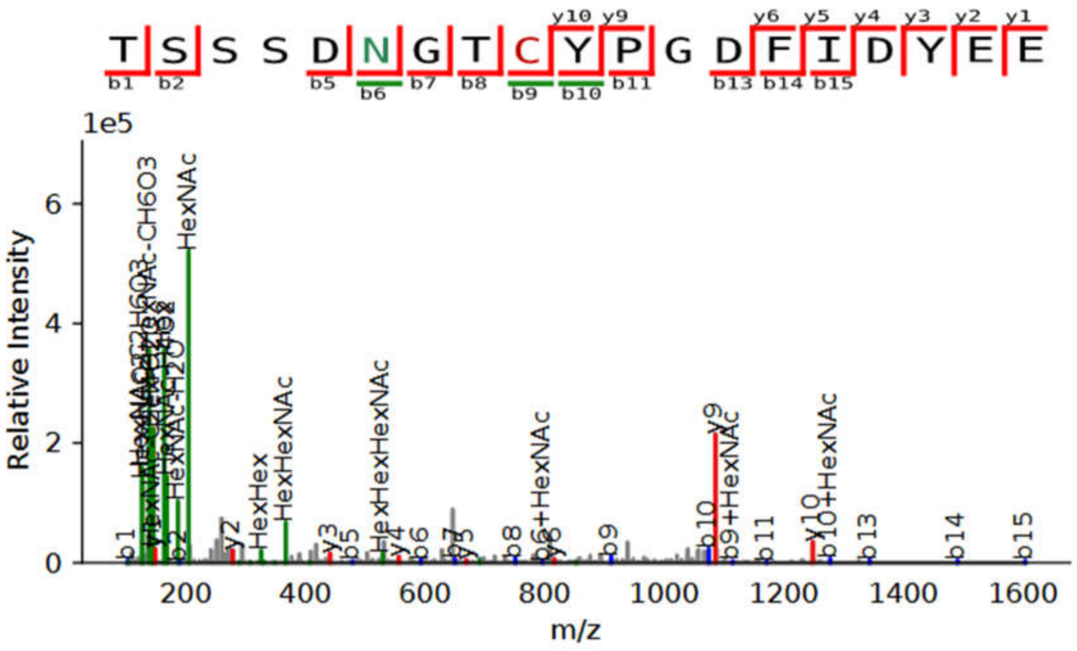

Figure S5. Annotated MSMS spectrum of M1 H1sD2 peptide TSSSD**N**GTCYPGDFIDYEE with Man10 glycan

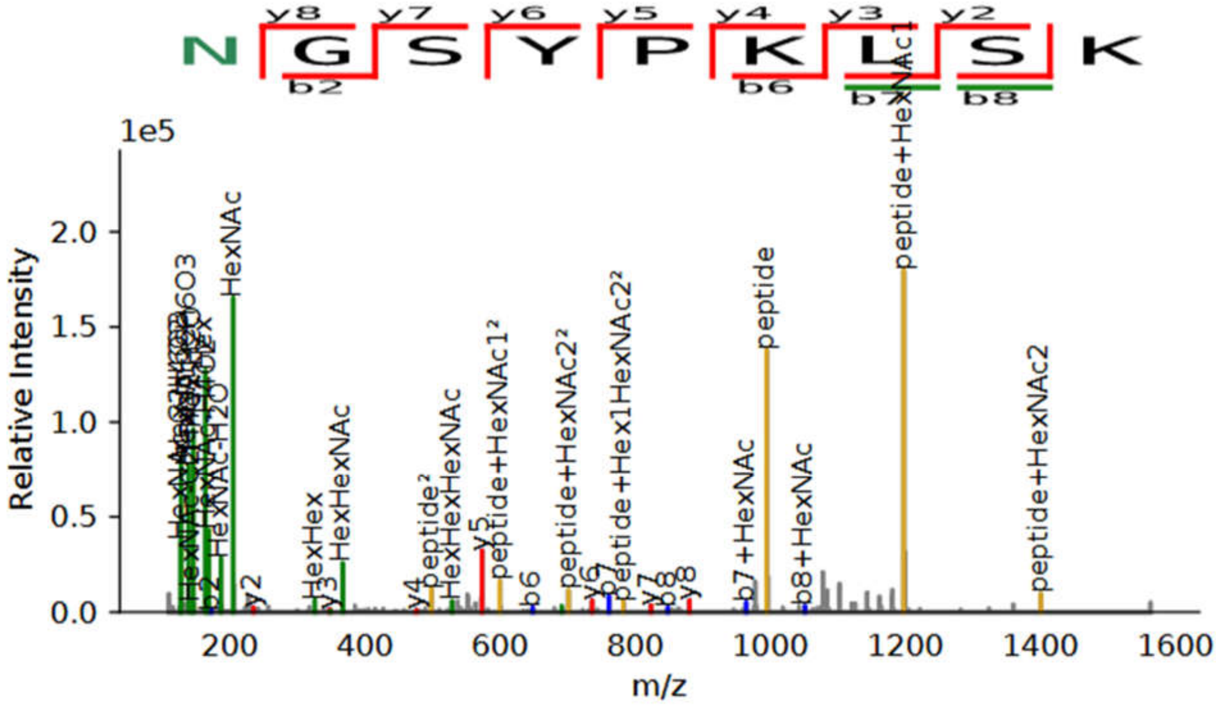

Figure S6. Annotated MSMS spectrum of M1 H1sD2 peptide **N**GSYPKLSK with Man11 glycan

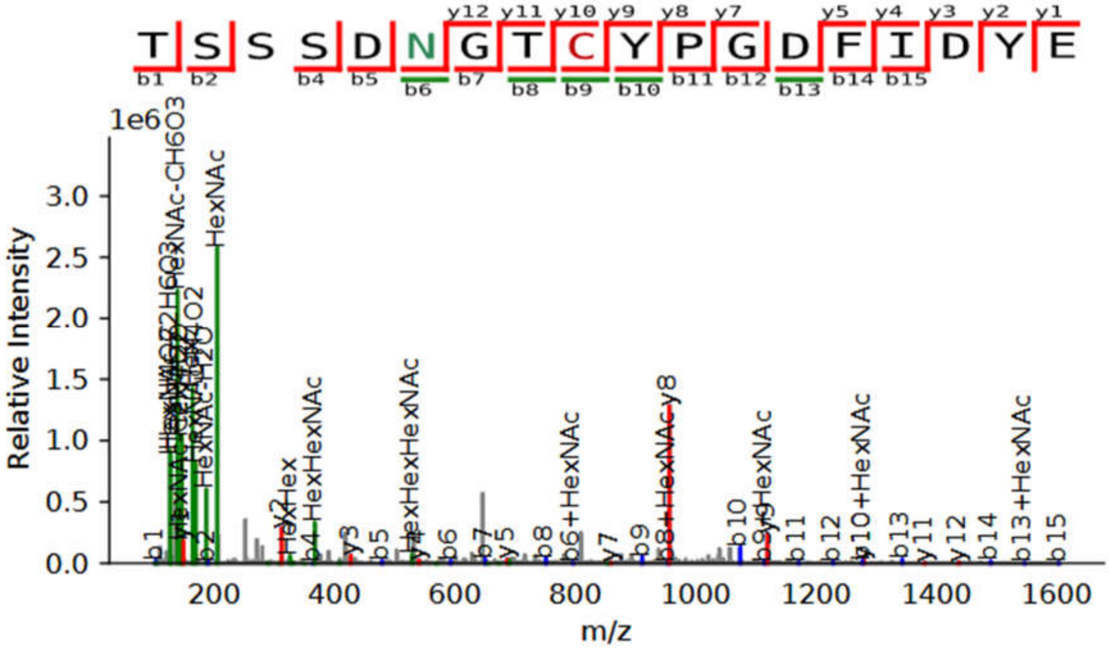

Figure S7. Annotated MSMS spectrum of M2 H1sD2 peptide TSSSD**N**GTCYPGDFIDYE with Man9 glycan

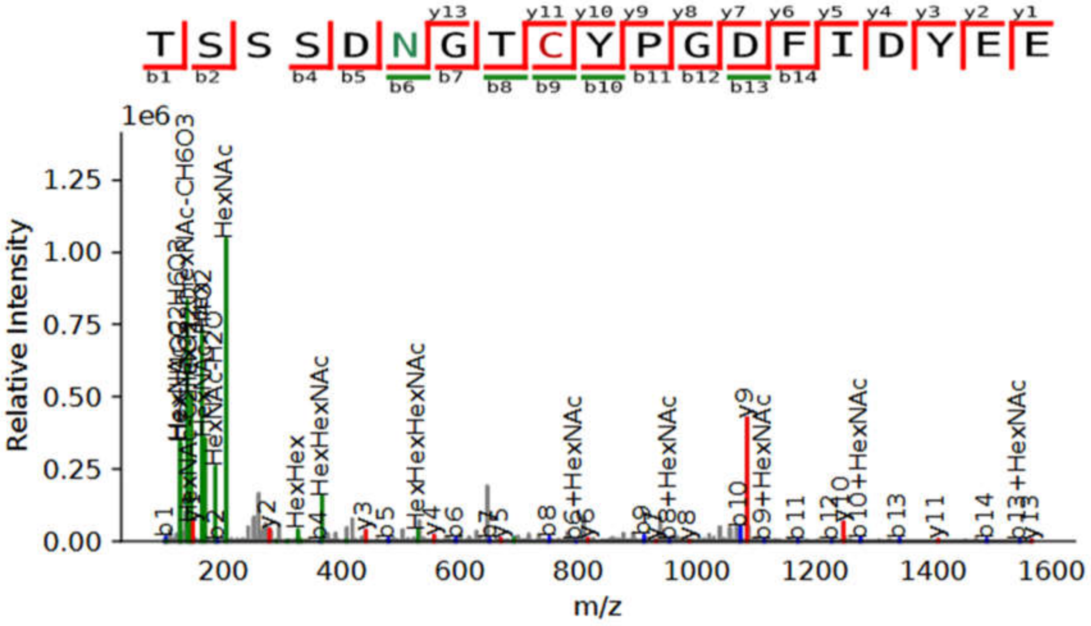

Figure S8. Annotated MSMS spectrum of M2 H1sD2 peptide TSSSD**N**GTCYPGDFIDYEE with Man10 glycan

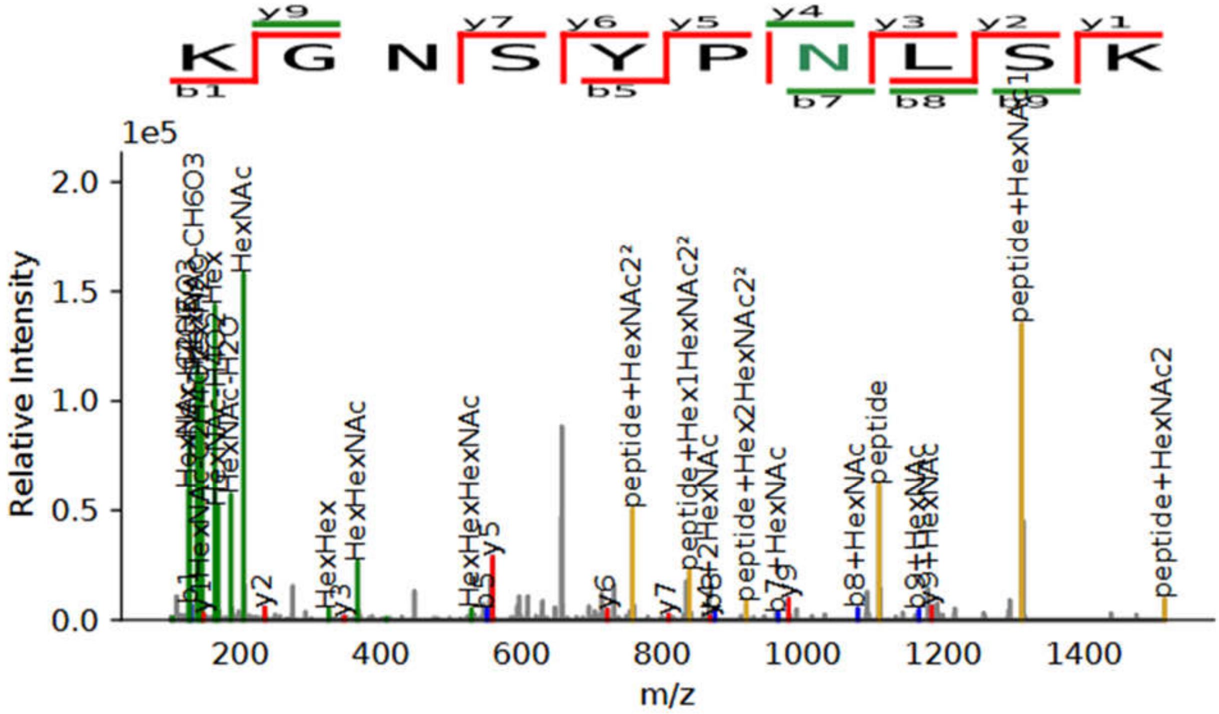

Figure S9. Annotated MSMS spectrum of M2 H1sD2 peptide KGNSYP**N**LSK with Man11 glycan

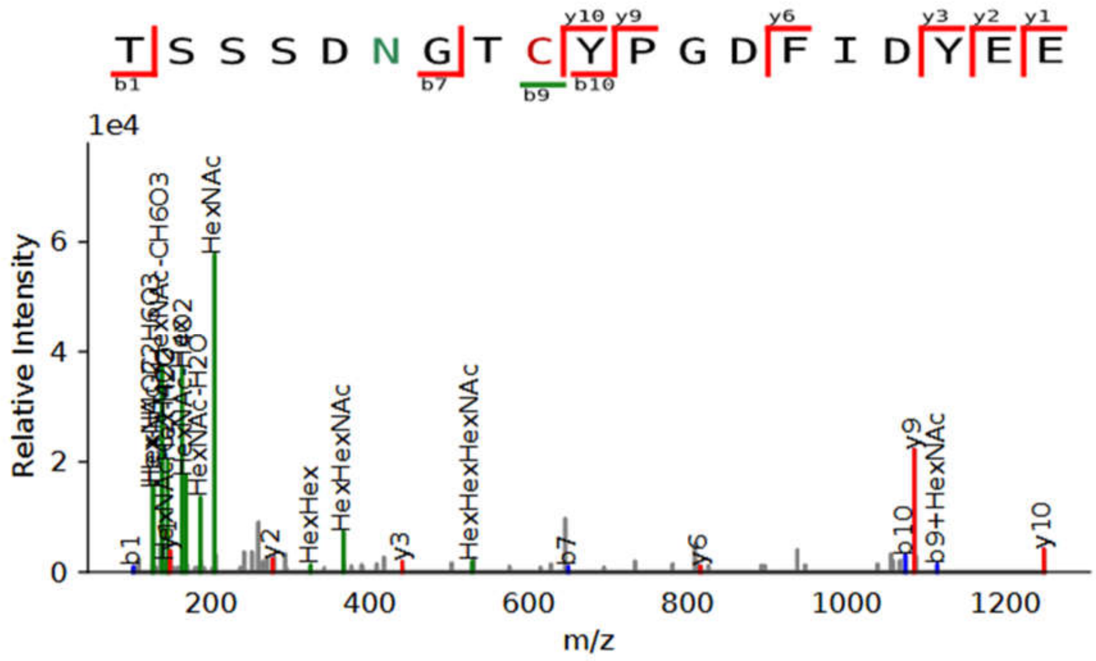

Figure S10. Annotated MSMS spectrum of M3 H1sD2 peptide TSSSD**N**GTCYPGDFIDYEE with Man9 glycan

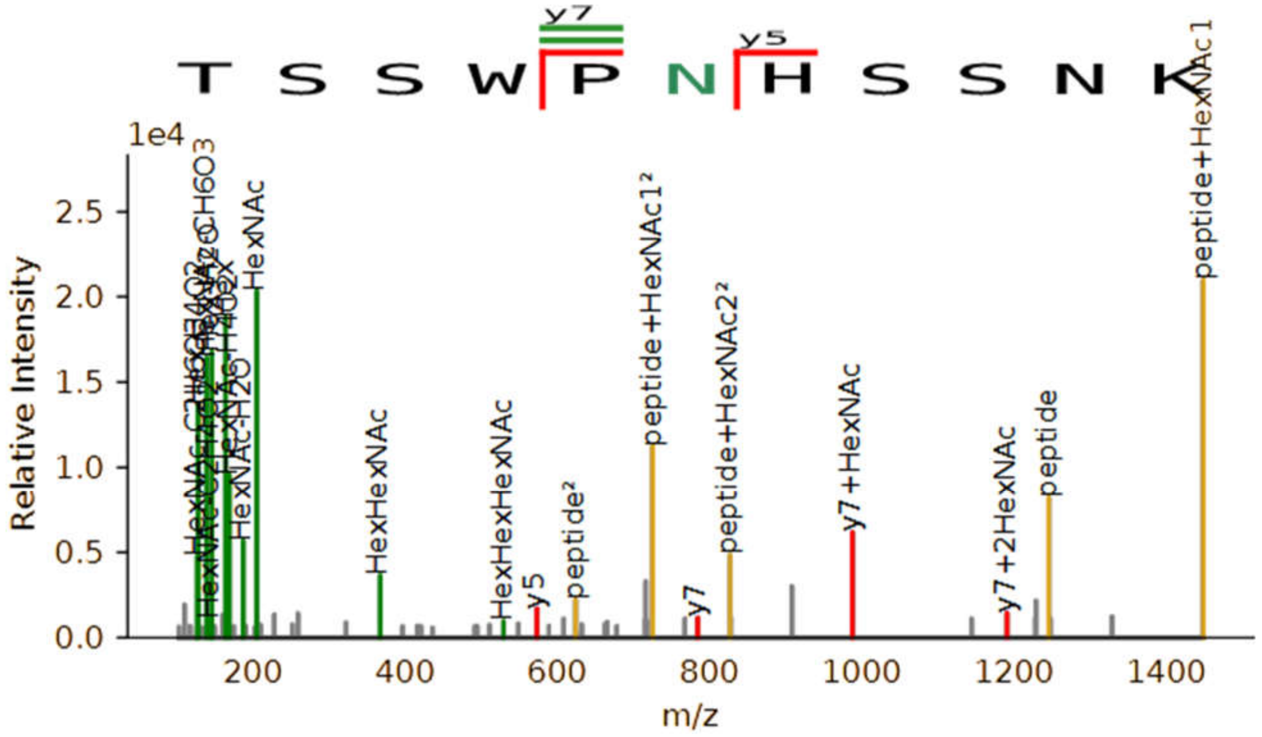

Figure S11. Annotated MSMS spectrum of M3 H1sD2 peptide TSSWP**N**HSSNK with Man9 glycan

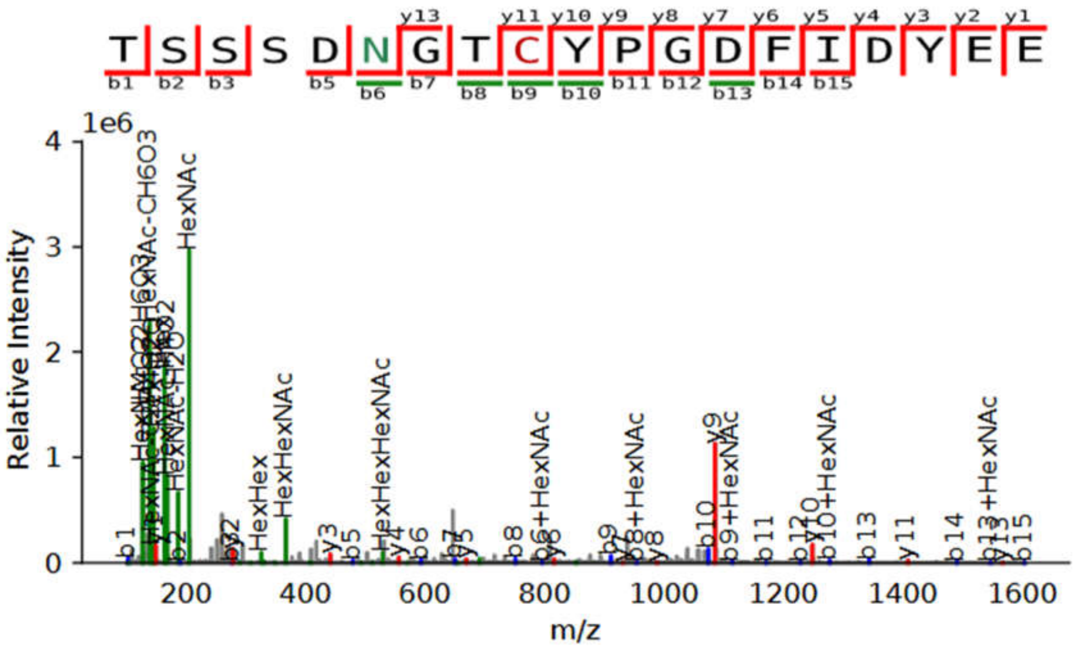

Figure S12. Annotated MSMS spectrum of M4 H1sD2 peptide TSSSD**N**GTCYPGDFIDYEE with Man10 glycan

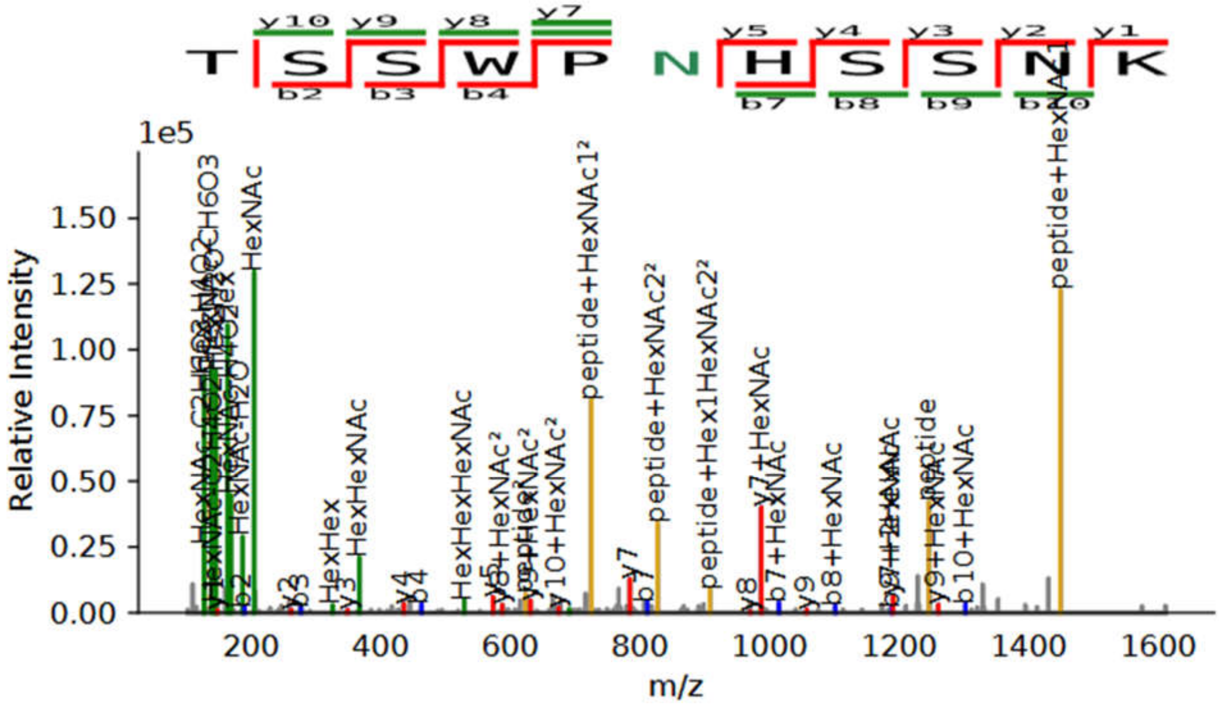

Figure S13. Annotated MSMS spectrum of M4 H1sD2 peptide TSSWP**N**HSSNK with Man10 glycan

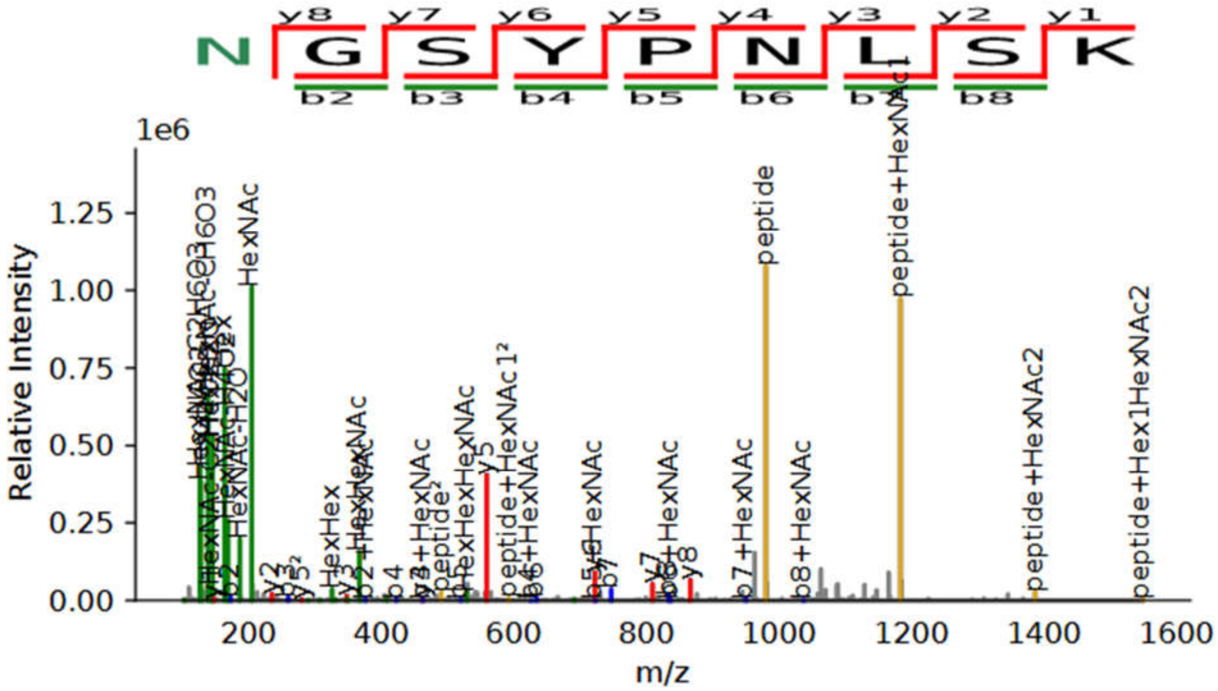

Figure S14. Annotated MSMS spectrum of M4 H1sD2 peptide **N**GSYPNLSK with Man10 glycan

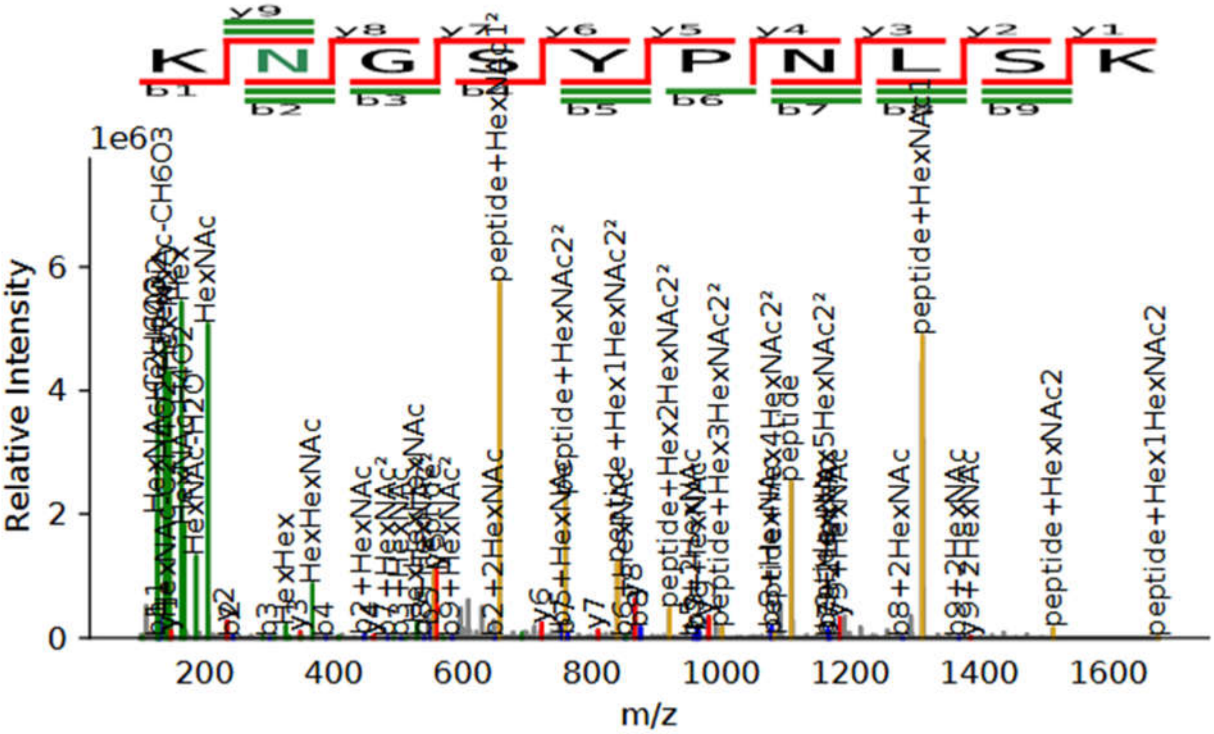

Figure S15. Annotated MSMS spectrum of M4 H1sD2 peptide K**N**GSYPNLSK with Man11 glycan

Confocal microscopy analysis

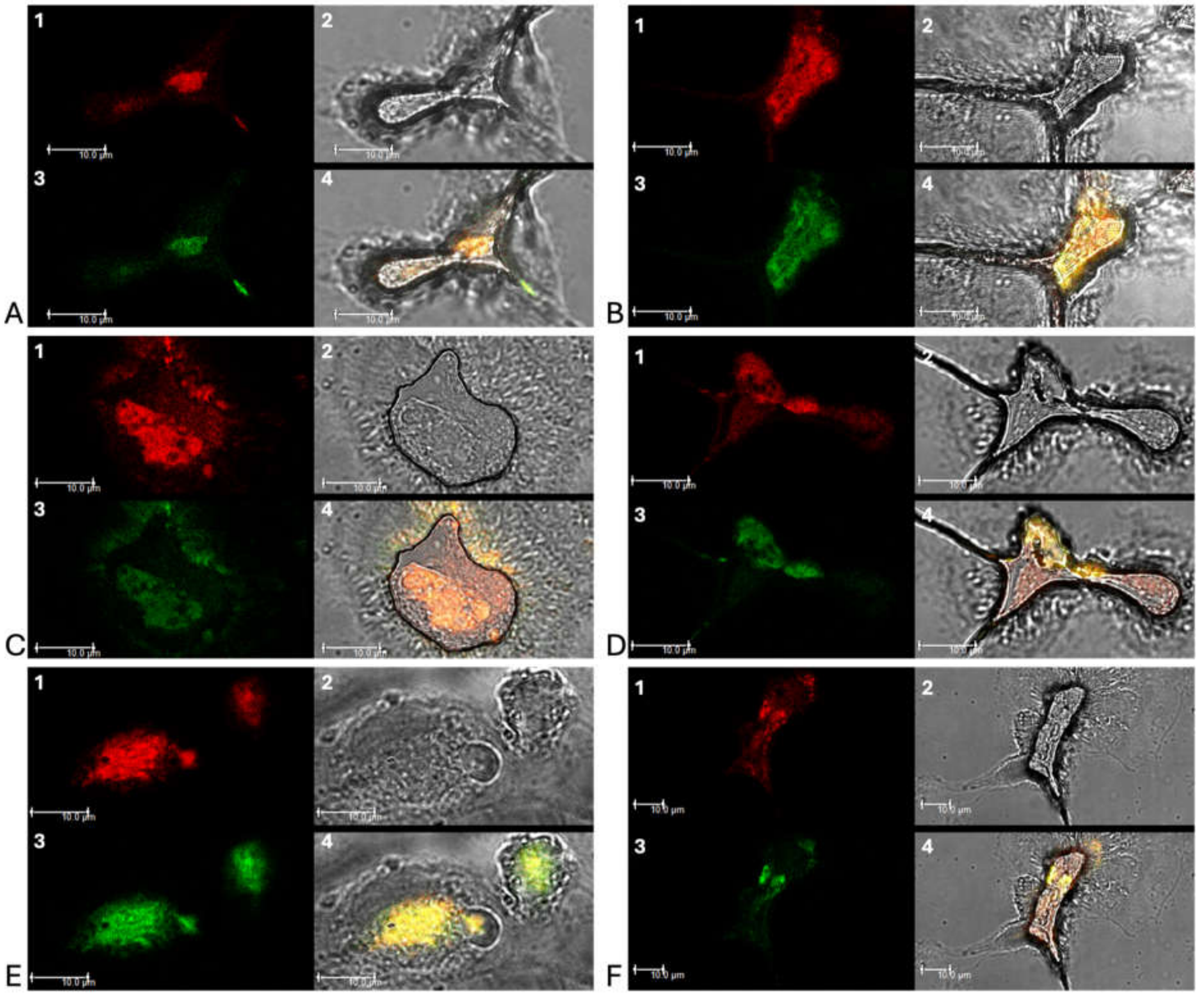

Figure S16. Confocal microscopy analysis of double-stained M2 macrophages with FITC-labeled H1sD2 and anti-DC-SIGN antibody. **A**) Der p 2; **B**) WT H1sD2; **C**) M1 H1sD2; **D**) M2 H1sD2; **E**) M3 H1sD2; **F**) M4 H1sD2. 1) PerCP- Cy5.5 (DC-SIGN); 2) TEM; 3) FITC (Der p 2 and H1sD2), 4) Overlay. In red, the localization of DC-SIGN receptors with anti-DC-SIGN antibody labeled with PerCP-Cy5.5 is presented. In green, the localization of hybrid H1sD2 glycoproteins after binding to surface receptors on M2 macrophages is presented. All images were captured at 64× objective magnification, with a scale bar indicating 10 µm.

Analysis of protein molecular weights by ProtParam and densitometry

Table S1. Theoretical and experimental determination of protein molecular masses, calculated from amino acid sequences (ProtParam) and estimated from SDS-PAGE band positions by densitometric analysis (ImageJ). R- reduced.

Protein	Theoretical Mw (kDa)	Experimental Mw (kDa)	
		SDS-PAGE R	SDS-PAGE R + PNGase F
**Der p 22**	15,07	16,9	16,9
**WT H1sD2**	40,88	49,4	45,9
**M1 H1sD2**	40,88	50,8	47,2
**M2 H1sD2**	40,87	51,9	47,4
**M3 H1sD2**	40,85	51,8	47,2
**M4 H1sD2**	40,84	52,9	48,5

Endotoxin determination

Table S2. Endotoxin determination in protein samples using LAL assay

Endotoxin	
**Protein**	**Concentration**
Der p 2	0.13 ng/mL
WT H1sD2	0.31 ng/mL
M1 H1sD2	0.28 ng/mL
M2 H1sD2	0.29 ng/mL
M3 H1sD2	0.16 ng/mL
M4 H1sD2	0.22 ng/mL

Patient serum and blood samples

Table S3. Clinical phenotypes of study participants

No.	Sex	sIgE HDM (kU/L)	Symptoms
1.	Male	d1: 0.43 class 1	allergic rhinitis
2.	Male	d1: 22.8 class 4	allergic rhinitis
3.	Female	d1:11.1 class 2	bronchus obstruction, asthma
4.	Male	d1: 0.56 class 1	allergic rhinitis, asthma
5.	Female	d1: 79.1 class 5	allergic rhinitis, asthma
6.	Female	d1: 54.7 class 5	allergic rhinitis
7.	Male	d1: 115.3 class 6	allergic rhinitis
8.	Male	d1: 1.57 class 2	allergic rhinitis
